# Increased expression of *ANAC017* primes for accelerated senescence

**DOI:** 10.1093/plphys/kiab195

**Published:** 2021-04-29

**Authors:** Martyna Broda, Kasim Khan, Brendan O’Leary, Adriana Pružinská, Chun Pong Lee, A Harvey Millar, Olivier Van Aken

**Affiliations:** 1 ARC Centre of Excellence in Plant Energy Biology, School of Molecular Sciences, University of Western Australia, Perth, Western Australia 6009, Australia; 2 Department of Biology, Lund University, Lund 22362, Sweden

## Abstract

Recent studies in Arabidopsis (*Arabidopsis thaliana*) have reported conflicting roles for NAC DOMAIN CONTAINING PROTEIN 17 (ANAC017), a transcription factor regulating mitochondria-to-nuclear signaling, and its closest paralog NAC DOMAIN CONTAINING PROTEIN 16 (ANAC016), in leaf senescence. By synchronizing senescence in individually darkened leaves of knockout and overexpressing mutants from these contrasting studies, we demonstrate that elevated *ANAC017* expression consistently causes accelerated senescence and cell death. A time-resolved transcriptome analysis revealed that senescence-associated pathways such as autophagy are not constitutively activated in *ANAC017* overexpression lines, but require a senescence-stimulus to trigger accelerated induction. *ANAC017* transcript and ANAC017-target genes are constitutively upregulated in ANAC017 overexpression lines, but surprisingly show a transient “super-induction” 1 d after senescence induction. This induction of *ANAC017* and its target genes is observed during the later stages of age-related and dark-induced senescence, indicating the ANAC017 pathway is also activated in natural senescence. In contrast, knockout mutants of *ANAC017* showed lowered senescence-induced induction of ANAC017 target genes during the late stages of dark-induced senescence. Finally, promoter binding analyses show that the *ANAC016* promoter sequence is directly bound by ANAC017, so ANAC016 likely acts downstream of ANAC017 and is directly transcriptionally controlled by ANAC017 in a feed-forward loop during late senescence.

## Teaser

Increased expression of a transcription factor that mediates mitochondrial signalling primes plants for accelerated senescence, induction of autophagy and cell death

## Introduction

Plants lead sedentary lifestyles in their vegetative growth phase and must therefore continuously adapt to changes in the environment, including abiotic or biotic stresses. Complex networks of gene expression underlie these stress responses (Coolen et al., 2016; Zhang et al., 2019). Intracellular signaling pathways are often divided into two main types as follows: anterograde signaling, when information originates from the nucleus and is transferred to other compartments of the cell (e.g. chloroplasts or mitochondria); and retrograde signaling which originates in the autonomous organelles and is transferred to the nucleus (Woodson and Chory, 2008). This tight communication between organelles is important for proper maintenance of cellular homeostasis and stress responses (Kwasniak et al., 2013; Jones, 2019; Wu et al., 2019). To study such signaling pathways, researchers have used naturally occurring triggers (e.g. light intensity) to induce specific responses, as well as chemical inhibitors and genetic approaches. Commonly used inhibitors that trigger chloroplast or mitochondrial retrograde signaling are paraquat, also known as methyl viologen, or antimycin A (AA), respectively.

Such studies have led to the identification of transcription factors and other proteins that are involved in retrograde signaling. More specifically for plant mitochondria-to-nucleus or chloroplast-to-nucleus signaling, a group of NAC domain containing transcription factors, including ANAC017 and ANAC013, was shown to play an important role during chemical inhibition of mitochondrial and chloroplast function (De Clercq et al., 2013; Ng et al., 2013; Van Aken et al., 2016a, 2016b). It was also determined that ANAC017 plays a role during developmentally controlled mitochondrial retrograde signaling (Ng et al., 2013; Van Aken et al., 2016a, 2016b). ANAC017 is localized in the endoplasmic reticulum (ER) membrane, and upon treatment for example with AA, the N-terminal end of ANAC017 is cleaved from the membrane and is thought to translocate to nucleus. There it recognizes a CT[T/C]GXXXXXCA[A/C]G-related motif (Ng et al., 2013; O'Malley et al., 2016) in promoters of stress responsive genes and regulates their expression (Ng et al., 2013). Knockout mutants of ANAC017 show strongly repressed mitochondrial retrograde signaling, which can be compensated during later stages (starting around 9-h post stress induction) by other transcription factors like ANAC013 (Ng et al., 2013; Van Aken et al., 2016a, 2016b). A recent study showed that a nuclear protein, RADICAL-INDUCED CELL DEATH1 (RCD1), interacts with and represses ANAC013 and ANAC017, thereby regulating both mitochondrial and chloroplast communication with the nuclear transcriptional apparatus (Shapiguzov et al., 2019). Interestingly, overexpression of ANAC017 in gain-of-function mutants leads to increased resistance to ER-stress (Chi et al., 2017).

An involvement of ANAC017 in plant senescence has also been proposed, but its exact role and its connection to retrograde signaling of organelle function remains unclear. Two recent independent studies have reported opposing developmental senescence phenotypes of ANAC017 mutants. Kim et al. (2018) showed that an *ANAC017* overexpressing line displayed a slower rate of leaf senescence, while knockout mutants exhibited a faster senescence phenotype, which suggests that ANAC017 is a negative regulator of natural senescence. On the contrary, overexpression of *ANAC017* reported by Meng et al. (2019) resulted in faster developmental leaf senescence than in control plants, which suggests a positive role of ANAC017 in senescence. *ANAC017* (*At1g34190*) is a neighboring and highly similar gene to *ANAC016* (*At1g34180*) and several studies have compared their contrasting role in dark-induced leaf senescence (Kim et al., 2013; Sakuraba et al., 2015, 2016). ANAC016 was described as a positive regulator of leaf senescence based on dark-induced senescence assays of detached leaves. *Anac016* knockout mutants retained dark green color and appeared to be healthy, while OE lines were heavily senescing after 4 d in darkness compared to wild-type (WT) (Kim et al., 2013). In the same study, dark-induced senescence of the *anac017* knockout mutant showed the same rate of senescence as WT plants and it was reported that ANAC017 did not play a role in senescence (Kim et al., 2013). Thus, far the ambiguity between these articles has not been addressed and remains unresolved. Delayed senescence can result in higher crop yield, while premature senescence phenotypes have been shown to reduce the yield in crops (Gregersen et al., 2013; Ma et al., 2018; Borrill et al., 2019). Therefore, unraveling the true role of ANAC017 in senescence could potentially be used in the selective breeding of crop plants.

In this study, we used a different experimental approach to determine the role of ANAC017 in plant senescence. Three main approaches to study plant senescence are commonly used in the literature: (1) following the natural senescence process of ageing leaves, (2) detaching healthy leaves from a non-senescent plant and keeping them in dark but humidified conditions to avoid dehydration, which assesses survival, and (3) individually darkening leaves that are still fully attached to a healthy plant (van der Graaff et al., 2006). By darkening only individual attached leaves, while keeping the rest of the plant in optimal conditions for plant growth, we analyzed dark-induced senescence in a synchronized way, while maintaining systemic communication, mimicking partial shadowing by a neighboring plant. By comparing the different ANAC017 mutants that were used in multiple studies (this study; Van Aken et al., 2016a, 2016b; Kim et al., 2018; Meng et al., 2019), together with *anac016-2* (Kim et al., 2013) we provide clear evidence that overexpression of ANAC017 positively regulates leaf senescence. Using a time-course approach in individually darkened leaves coupled with RNA-seq and RT-qPCR, we are able to provide detailed information on the expression response of ANAC017 targets and the rate of senescence in different lines. Promoter binding assays further show that ANAC017 directly binds the *ANAC016* promoter and the observed expression patterns indicate that ANAC017 co-regulates the expression of *ANAC016*.

## Results

### Overexpression of ANAC017 leads to increased senescence rate

**Figure 1:**
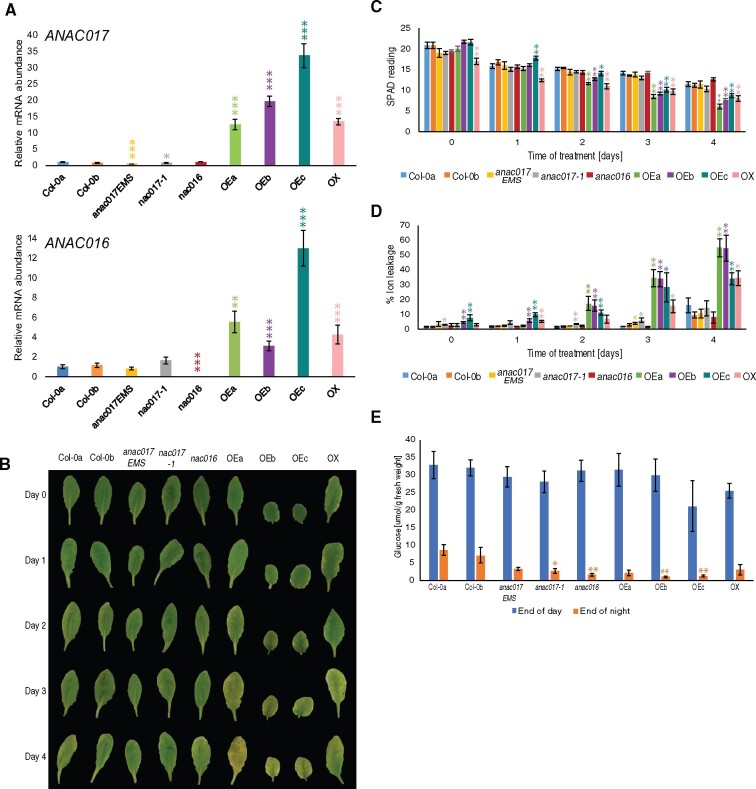
Overexpression of ANAC017 leads to accelerated senescence. A, Expression levels of *ANAC017* and *ANAC016* in a range of *ANAC017* knockout and overexpressing genotypes determined by RT-qPCR. B, Phenotype of analyzed genotypes in individually darkened leaf senescence time-course at indicated time points. For representation purposes, leaves were digitally extracted and placed on a black background. C, Chlorophyll measurements during senescence time-course at indicated time points. D, Ion leakage measurements taken during dark-induced senescence time-course. E, Starch levels presented as the glucose equivalents at end of day and end of night (*n* = 6). Student’s *t* test was used to simplify visualization of statistical differences between WT and each genotype at a given time point. *P < 0.1, ** *P* < 0.05, ***P < 0.01, full two-way ANOVA results are shown in Supplemental Figure S3. Error bars indicate standard error.

To resolve the disputed senescing phenotypes of different *ANAC017* knockout mutants and overexpressing lines we collected mutants from several studies, including *anac017EMS* generated by EMS mutagenesis, SALK T-DNA insertion mutant *anac017-1* (Ng et al., 2013), and 35S CaMV overexpression lines *ANAC017 OEa* (Van Aken et al., 2016a, 2016b) and *ANAC017 OX* (Kim et al., 2018). We also produced additional independent overexpression lines *ANAC017 OEb-c* (Figure 1, A and B). ANAC016 has been published previously as a positive regulator of senescence (Kim et al., 2013; Sakuraba et al., 2015, 2016). The *ANAC016* gene (*At1g34180*) is located next to *ANAC017* (*At1g34190*) on the Arabidopsis (*Arabidopsis thaliana*) genome and is the closest paralog to ANAC017, with 71% sequence identity (Supplemental Figure S1A). We therefore tested the role of ANAC016 during retrograde signaling responses. We used AA as a mitochondrial stress inducer on *anac016* knockout plants and tested the expression of *Alternative Oxidase 1a* (*AOX1a*), a classical marker for mitochondrial stress responses. We were able to show that retrograde signaling responses are no different in *anac016* knockout mutants compared to Col-0a plants, while the *anac017EMS* knockout mutant displays almost complete abolishment (Supplemental Figure S1B).

We tested all lines used in the study for the gene expression levels of *ANAC017* (Figure 1A). Consistent with previously published findings, knockout mutants of *ANAC017* had less transcript, with higher significance in the *anac017EMS* mutant line compared to Col-0a plants. In line with displaying the strongest growth defects, highest overexpression of *ANAC017* was observed in *OEb* and *OEc* lines (20- and 34-fold change, respectively), which display a similar phenotype as *OE2* and *OE3* published by Meng et al. (2019). Since ANAC016 was previously published as a positive regulator of senescence, we also analyzed its transcript level in 5-week-old plants. *ANAC016* transcript was undetectable in *anac016* mutant plants. Interestingly, *ANAC016* has increased expression in all the ANAC017 overexpression lines examined (Figure 1A), suggesting it is positively regulated by ANAC017.

**Figure 2:**
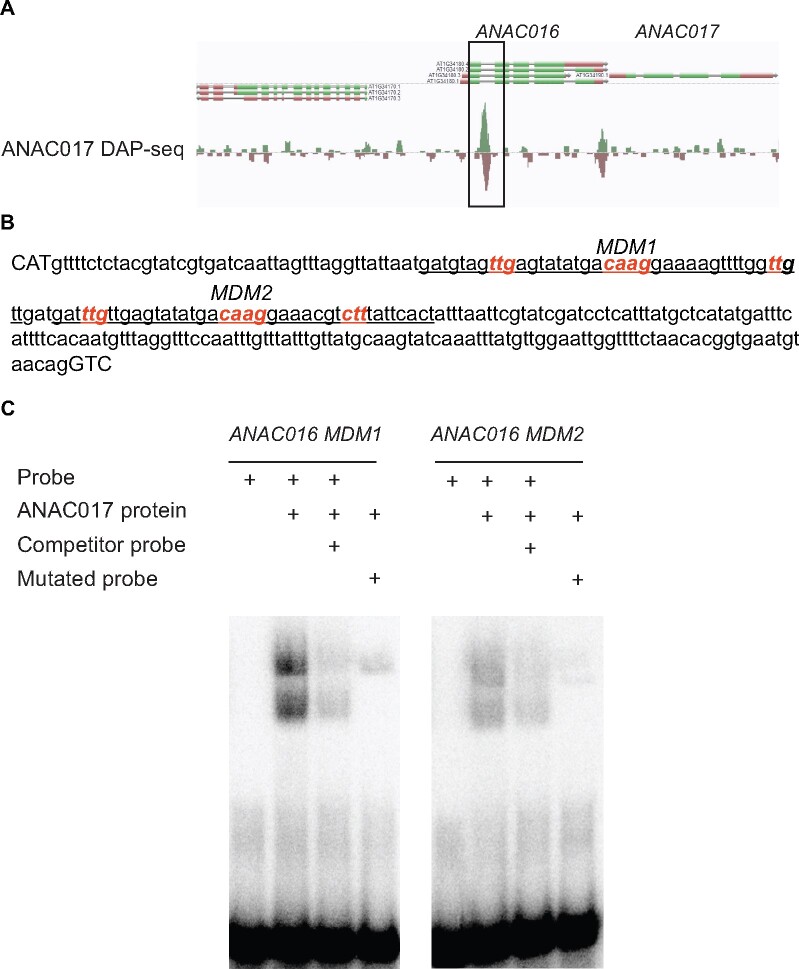
ANAC017 binds *ANAC016* regulatory promoter sequences. A, ANAC017 binding site in the first intron of *ANAC016* as identified by DAP-seq analysis (O'Malley et al., 2016). B, DNA sequence of the first *ANAC016* intron (lower case letters) flanked by three bases of exon 1 and 2 (uppercase letters). The two MDM probe regions used for the EMSA’s are underlined. The main CAAG motif and flanking reverse complement-like sequences are indicated in bold italics. The bases that were altered for the mutated probes are indicated in red. C, EMSAs showing direct binding of ANAC017 to putative MDM in the *ANAC016* promoter sequence (intron 1) as indicated by the DAP-seq analysis in (A). Radiolabeled dsDNA probes spanning the MDM sites were separated on acrylamide gels. Purified recombinant ANAC017 lacking the C-terminal transmembrane domain was added in marked lines. Unlabeled competitor probe was added in lane three. In the fourth lane, radiolabeled dsDNA probes were loaded in which the MDM sites were mutated.

We therefore considered if the expression of *ANAC016* could be regulated by ANAC017 or vice versa. ANAC016 has been found in one study to bind a motif that is nearly identical to a motif bound by ANAC017, C[TG]TGXXXXXCA[A/C]GXA (O'Malley et al., 2016; Supplemental Figure S2A), which is termed the mitochondrial dysfunction motif (MDM) (Supplemental Figure S2B; De Clercq et al., 2013). However, a different study reported that ANAC016 binds to a completely different “ANAC016 binding motif” (ANAC16BM; Sakuraba et al., 2015), GATTGGAT[A/T]CA (Supplemental Figure S2C). Neither ANAC16BM nor clear MDM motifs are found in the promoter region of ANAC017. We therefore verified if ANAC017 bound the *ANAC016* promoter in a previously published Arabidopsis DNA affinity purification sequencing (DAP-seq) experiment (O'Malley et al., 2016). This revealed that ANAC017 showed a clear and significantly enriched binding site in the first intron of *ANAC016* (Figure 2A). First, introns have previously been shown to contain important information for correct transcription (Rose, 2018; Gallegos and Rose, 2019). The *ANAC016* first intron region contains two putative ANAC017-binding motifs with a core CAAG sequence (Figure 2B; Ng et al., 2013; O'Malley et al., 2016). To verify that these are bona fide ANAC017 binding sites, we purified recombinant ANAC017 without the C-terminal transmembrane domain and performed electromobility shift assays (EMSAs) using two 40-bp radiolabeled probes that span one putative MDM motif (Figure 2, B and C). A very clear shift in mobility was observed after addition of recombinant ANAC017 for both probes, with the first motif (MDM1) showing the strongest binding. To test the specificity of the binding, we added unlabeled competitor probe, which clearly reduced the binding of the radiolabeled probe. Finally, we specifically mutated the putative MDM sites in these probes, which almost completely abolished the mobility shift (Figure 2, B and C), indicating ANAC017 directly and specifically binds the *ANAC016* gene sequence. Together, the increased expression of *ANAC016* in *ANAC017* overexpression lines (Figure 1A) and DAP-seq/EMSA results (Figure 2) suggest that *ANAC016* is a target for expression regulation by ANAC017.

We then analyzed the leaf senescence phenotype in individually darkened leaves 6 and 7 of 5-week-old plants grown under long-day (LD) conditions at Days 0, 1, 2, 3, and 4 post treatment. Three out of four *ANAC017* overexpressing lines (*OEa-OEc*) displayed visible yellowing of leaves already at Day 2 compared to both Col-0 controls (Figure 1B). The fourth overexpression line, *ANAC017 OX* (Kim et al., 2018), displayed senescence a day later. All *ANAC017* overexpression lines had visibly wilted by Day 4. In contrast, both *anac017* and *anac016* knockout mutants remained green and retained cell integrity, with no visible differences to Col-0a and Col-0b plants over the full senescence time-course. To quantitatively characterize, the differences in those lines, we analyzed the level of chlorophyll and ion leakage across the cell membrane. Two-way ANOVA analysis showed time-dependent differences for both metrics within all analyzed genotypes, however no genotype-dependent differences were observed between *anac017*EMS and *anac016* knockout mutants compared to WT plants (Figure 1, C and D). *anac17-1* showed a mild but significant basal increase in ion leakage compared to Col-0 during the first three days of the time course. A genotype and genotype:time effect in chlorophyll content and ion leakage was confirmed for all overexpressing lines compared to Col-0a and Col-0b plants (Figure 1C;Supplemental Figure S3). Overexpression of *ANAC017* resulted in a significant decrease in chlorophyll content in all overexpression lines by Day 2, and this difference with Col-0 plants increased further at later stages of the senescence time-course (Figure 1, B and C). Consistent with the loss of chlorophyll, ion leakage of cell membranes increased dramatically in all four OE lines over the time course (Figure 1D). To assess whether *ANAC017 OE* also resulted in accelerated senescence using a detached leaf assay, we collected leaf 6–7 from 5-week-old plants and placed them in moistened petri dishes in the dark. Clear visible signs of senescence were observed in the *ANAC017 OE plants* already at 2 days in darkness, and visibly chlorotic and wilted leaves were apparent after three days in the dark, while detached Col-0 and *anac017EMS* leaves maintained a healthy appearance even after three days in the dark (Supplemental Figure S4).

**Figure 3:**
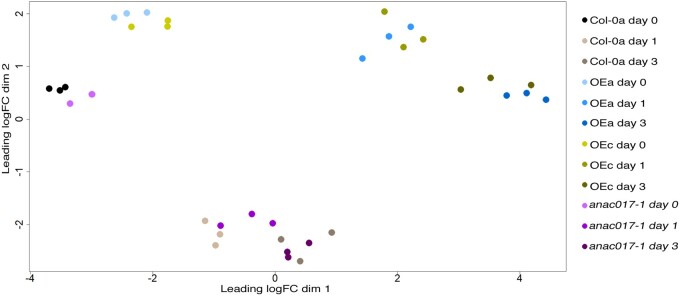
Exploration of RNA-seq data from dark-induced senescence assay in Col-0a, *anac017-1*, *ANAC017 OEa*, and *OEc* plants. RNA-seq MDS plot of transcripts in four analyzed genotypes at Days 0, 1, and 3 of the senescence time-course. Each data point represents the relative overall distance between the RNA-seq samples. LogFC, Log2 fold change.

As effects of ANAC017 or ANAC016 loss of function may not become clear until after Day 4, we repeated the senescence assay on individually darkened leaves and extended the time course until Day 7 post senescence induction. Using a two-way analysis of variance (ANOVA) analysis, we found that there is no consistent genotype-dependent difference in visible senescence rate or chlorophyll loss between Col-0a and *anac016* or either *anac017* knockout lines used in the study (Supplemental Figure S5).

*ANAC017 OE* lines had previously been reported to contain lower levels of starch (Meng et al., 2019), but no quantitative data were provided in that study. It could be speculated that dysfunction in starch synthesis or degradation might affect senescence phenotypes in *ANAC017-*related genotypes. We tested the amount of starch in 5-week-old plants at the end of the day and end of night, which would reveal whether these genotypes have the same synthesis and degradation capability as Col-0 plants. Our results show that there is no difference in starch content between any of the lines at the end of the day (Figure 1E), which indicates that they were all competent in starch synthesis. Also, all lines were able to degrade the majority of their starch during a night period which indicates that they were competent in starch degradation. *Anac016* and *anac017-1* knockout and two out of four *ANAC017* overexpression plants had lower levels of starch at the end of the night than Col-0 (Figure 1E); however, based on these results, the accelerated senescence phenotype cannot be associated with the observed starch levels.

### Exploration of genome-wide transcriptional responses during leaf senescence

To further analyze how knocking-out or overexpressing *ANAC017* influences transcriptional responses, we performed RNA-seq analyses. For this, leaves 6 and 7 were pooled together and analyzed at Days 0, 1, and 3 of the previously described individually darkened leaf senescence time-course for Col-0a, *anac017-1* and two overexpressing lines *OEa* and *OEc*. *OEa* and *OEc* were chosen because they display contrasting developmental phenotypes (big and oval leaves in *OEa*, small and curly leaves in *OEc*) but both show accelerated senescence. Generally, transcript counts were mapped and a minimum of 21,243 transcripts were detectable in each of the 36 samples (Supplemental Table S1), with an average of about 24 million counts per sample (Supplemental Table S2). The multidimensional scaling (MDS) that reduces the complexity of the whole genome RNA-seq data revealed that the Col-0a and *anac017-1* transcriptomes could not be clearly distinguished from each other, while both overexpressing lines clustered together (MDS plot, Figure 3). In contrast, there was a much greater dissimilarity in the MDS plots between the overexpressor and Col-0a/*anac017-1* groupings, and the distance between the two groups increased further throughout the time-course (Figure 3). Within Col-0a, there were 8,263 differentially expressed genes (DEGs) after Day 1 of darkening, and 9,041 after Day 3 (Table 1). At Day 0 of the senescence time-course, *anac017-1* had in total 1,205 DEGs compared to Col-0a, the majority of which (714) were downregulated (Table 2). Much larger numbers of DEGs were obtained for the OE lines compared to Col-0a: 4,178 and 4,504 for *OEa* and *OEc*, respectively, similar to results obtained by (Meng et al., 2019; Table 2). However, the number of DEGs increased by almost three-fold and two-fold at Days 1 and 3 of the senescence time-course, respectively.

**Table 1:** Number of significantly changed transcripts in the RNA-seq dataset at indicated days of the senescence time-course and indicated comparisons in Col-0a

Comparison		Number of Changed Transcripts
Col-0a Day 1 versus Day 0	Upregulated	1,546
	Downregulated	6,717
Col-0a Day 3 versus Day 0	Upregulated	2,874
	Downregulated	6,167
Col-0a Day 3 versus Day 1	Upregulated	1,695
	Downregulated	975

**Table 2:** Number of significantly changed transcripts in the RNA-seq dataset at indicated days of the senescence time-course and indicated comparisons

Comparison		Day 0	Day 1	Day 3
*anac017-1* versus Col-0a	Upregulated	491	441	651
	Downregulated	714	283	227
*OEa* versus Col-0a	Upregulated	3,455	9,058	6,385
	Downregulated	723	1,030	3,188
*OEa* versus Col-0a	Upregulated	2,687	9,185	6,726
	Downregulated	1,817	1,378	2,352
*OEa* versus OEc	Upregulated	198	188	1,474
	Downregulated	1,150	269	389

**Figure 4:**
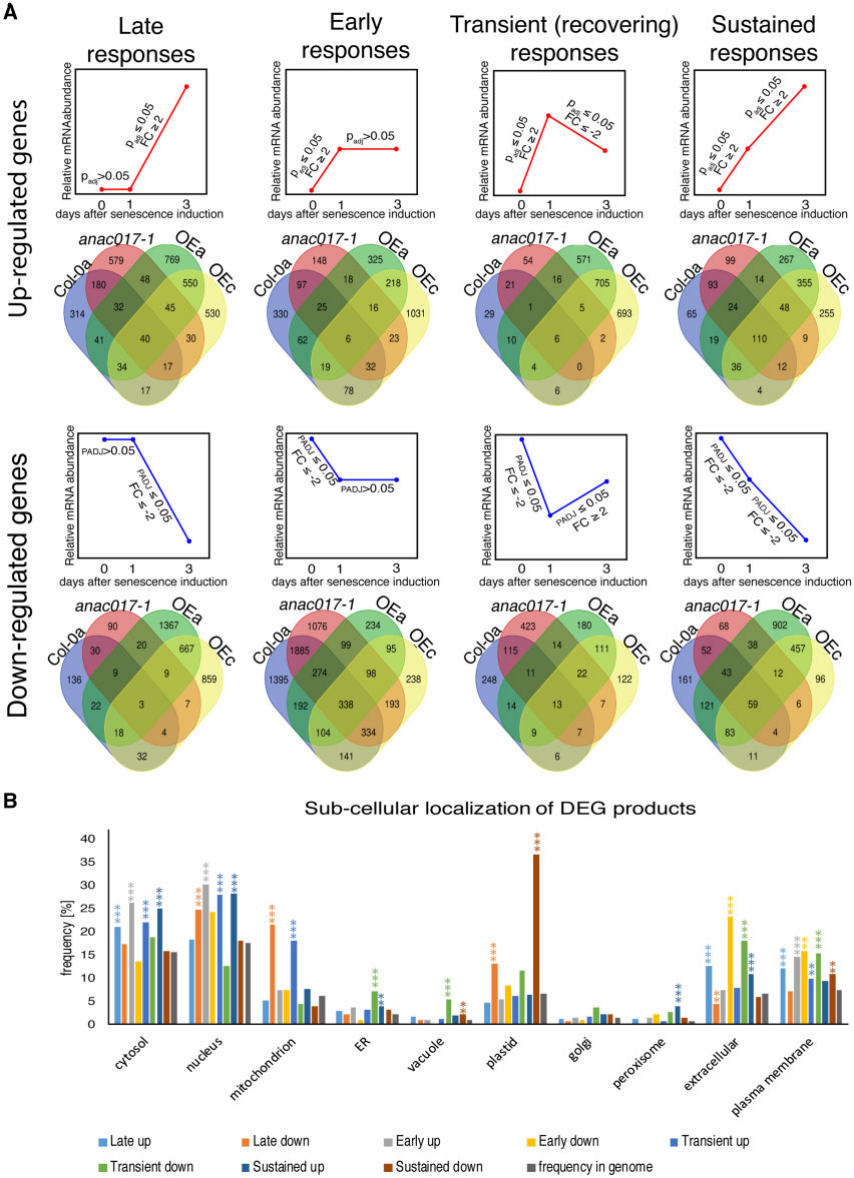
Time and localization-dependent transcriptional responses based on RNA-seq data. A, Four types of responses in RNA-seq dataset defined by *P*_adj_ value and fold change, presented on figures above appropriate Venn diagrams. Upregulated transcripts (top pane, red), downregulated transcripts (bottom pane, blue). B, Frequency of subcellular localization of DEG products within each type of response described above, common between *ANAC017 OEa* and *OEc*. Asterisks represents statistical significance (Fisher’s exact test) *P < 0.1, ** *P* < 0.05, ***P < 0.01.

We then determined if there are sets of genes that respond differently over time in the four tested genotypes. On the basis of the gene expression patterns, we divided the senescence responses into four types (represented as small graphs, Figure 4A): late responses, where transcripts were significantly upregulated or downregulated only at Day 3 post darkening; early responses, where transcripts were significantly elevated or decreased at Day 1, with no further change at Day 3 compared to Day 1; transient responses, where transcripts were upregulated or downregulated at Day 1, and at Day 3 post darkening the level of transcripts are recovering toward their original level; and finally sustained responses, where significantly changed transcripts were observed at Day 1 post treatment, and continued in the same direction on Day 3. The Venn diagrams show how the genes representing each of the four expression patterns are conserved between the different genotypes.

Next, we focused our attention to genes where transcription was significantly changed in both overexpressing lines in the same way. Transient type responses had the smallest number of genes with changed expression (877 genes). The majority of genes showed late type responses (1,366), followed by sustained (1,160), and early (894) responses (Figure 4). As senescence is known to impact differentially on the various subcellular compartments, such as chloroplasts and mitochondria (Keech et al., 2007), we analyzed whether products of DEGs (in those four groups were targeted to specific subcellular locations (Figure 4B). Large differences could be observed for transcripts encoding proteins located in the mitochondrion and plastid. Most of the DEGs encoding mitochondrial proteins belonged to late down and transient up groups, while DEGs encoding plastid proteins were mostly in the late down and sustained down responses.

**Figure 5:**
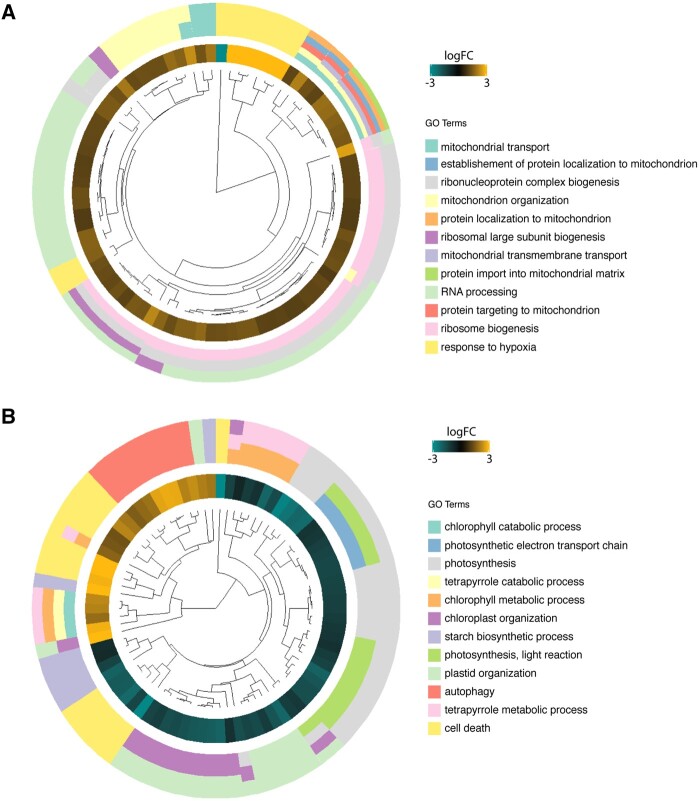
Enrichment of GO terms. A, Transient and (B) sustained type responses common between *ANAC017 OEa* and *OEc*. For visualization purposes (log2 transformed) fold change values represent the mean of *ANAC017 OEa* and *OEc* fold changes are shown in the inner concentric circles, while the outer concentric circles show GO terms associated with the same genes. The color scale bar represents average log 2-fold gene expression values for *ANAC017 OEa* and *OEc.*

We then widened our search and looked at gene ontology (GO) enrichment in genotype-specific groups (Supplemental Table S3) and identified clusters of GO terms that were specific to *transient* or *sustained* responses occurring in both ANAC017 OE lines (Supplemental Table S4). In *transient* responses, GO term enrichment was found for ribosomal biogenesis, RNA processing and mitochondrial function processes (mitochondrial transport and organization, import into mitochondria; Figure 5A); these GO terms represented 68% of all transiently expressed genes, and contained mostly upregulated genes. Sustained response gene sets displayed GO term enrichment for catabolism of tetrapyrrole and chlorophyll, photosynthetic processes and organization of chloroplasts (Figure 5B), which is consistent with results from subcellular localization studies. Most of the genes in these groups showed sustained downregulation.

### Autophagy and cell death-related genes become hyper-activated in *ANAC017 OE* lines after induction of senescence

**Figure 6:**
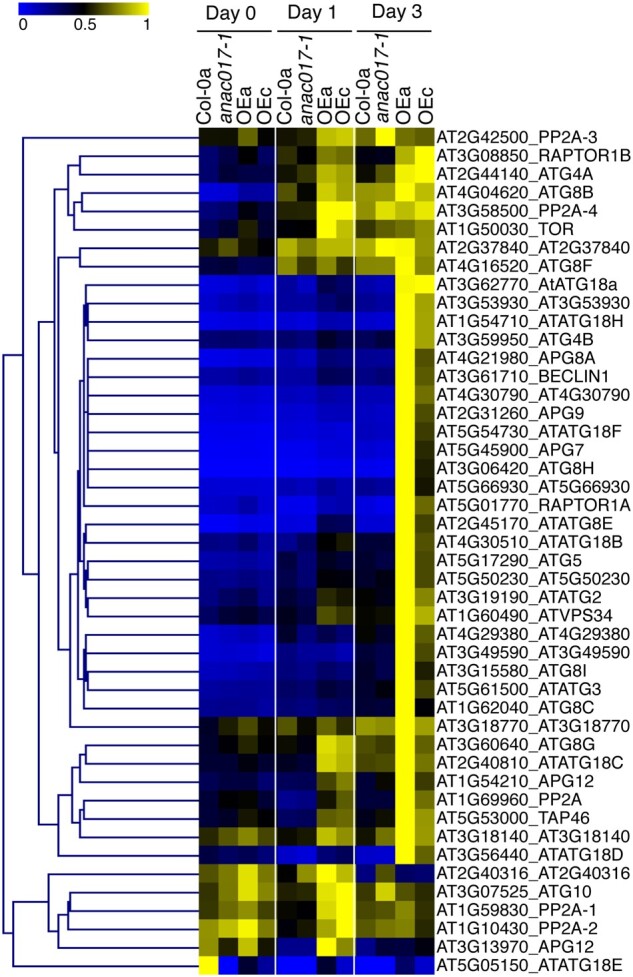
Heat map of DEGs that belong to the autophagy pathway, during the dark-induced senescence assay in ANAC017-related genotypes. Lowest and highest expression values for each gene have been normalized between 0 and 1.

Interestingly, DEGs with sustained upregulation in *ANAC017 OE* lines compared to Col-0 were enriched in GO terms for autophagy-related components and cell death. These results agree with the senescence assay showing that *ANAC017 OE* lines consistently senesce faster than Col-0, *anac017*, and *anac016* lines. We therefore looked for further evidence of transcriptional induction of the autophagy pathway. We were able to detect 46 of the key components of autophagosome regulation and formation (Thimm et al., 2004) in our RNA-seq data including *AuTophaGy* (*ATG*) genes and Target of Rapamycin kinase, with the exception of two genes: *LST8-2* (*At2g22040*), expressed only in silique and seeds, and *ATG8D* (*At2g05630*). In contrast to previous findings (Meng et al., 2019), autophagy-related genes (ATGs) were not constitutively upregulated in ANAC017 overexpressing lines before the induction of senescence (Day 0). However, they were induced much more strongly in both *ANAC017 OE* lines at Day 1 and/or Day 3 of the dark-treatment (Figure 6; Supplemental Table S1). In agreement, the GO category “pre-autophagosomal structure” was overrepresented among sustained UP genes in both ANAC017 OE lines (Supplemental Table S3). In conclusion, the ANAC017 OE lines have normal expression of ATG-related genes, but after 1 d of darkening, the ATG-pathway is strongly induced and continues to rise at Day 3.

### Hormone signaling is affected in ANAC017 mutants

Previous reports identified differential expression of genes related to jasmonic and/or salicylic acid hormone signaling, which are known to induce senescence, in *ANAC017* mutant plants (Kim et al., 2018; Meng et al., 2019). We therefore screened our RNA-seq data for genes associated with the metabolism and signal transduction of hormones that can induce senescence (SA, JA, and ethylene; Morris et al., 2000; He et al., 2002; Qiu et al., 2015) or restrain senescence (gibberellins, auxins, cytokinins; Xiao et al., 2015; Hu et al., 2017; Serova et al., 2019). Genes associated with metabolism and signal transduction of jasmonic acid, ethylene, and salicylic acid were significantly upregulated in *ANAC017 OE* lines compared to either Col-0a or *anac017-1* lines (Supplemental Figure S6A; Supplemental Table S1). Genes representing negative regulation of senescence through cytokinins, auxins, and/or gibberellins also seemed to be downregulated in all genotypes already after Day 1 of darkness, and in several cases, the downregulation was observable already at Day 0 in *ANAC017 OE* lines (Supplemental Figure S6A; Supplemental Table S1). Therefore, we further analyzed genes found downstream of hormonal pathways previously shown to be involved in senescence, including SENESCENCE ASSOCIATED GENES (SAGs), YELLOW-LEAF-SPECIFIC GENE 7 (YLS7), and YELLOW-LEAF-SPECIFIC GENE 9 (YLS9), PROTEIN ARGININE DEIMINASE 4 (PAD4; Yoshida et al., 2001; Vogelmann et al., 2012; James et al., 2018; Jin et al., 2018). The majority of those genes were upregulated over the senescence time-course either at Day 1 or 3 in both *ANAC017 OE* lines (Supplemental Figure S6B; Supplemental Table S1). This, together with the expression of genes associated with autophagy, cell death and metabolism of JA, SA, and/or ethylene is in agreement with faster senescence in *ANAC017 OE* lines.

### Core ANAC017-regulated genes are transiently “super-induced” during senescence in *ANAC017 OE* lines

**Figure 7:**
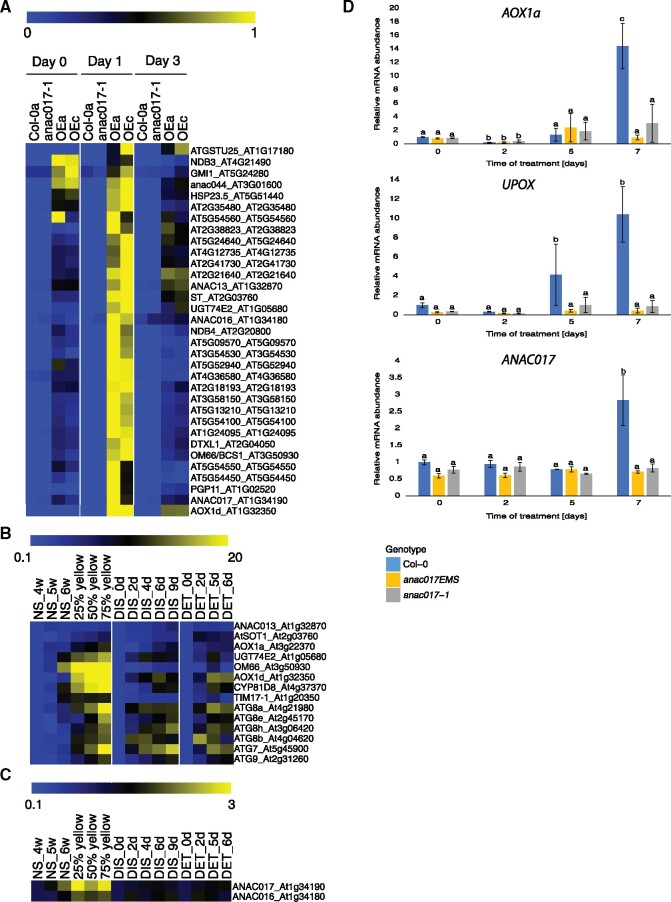
Core ANAC017-regulated genes are differentially expressed in dark-induced and natural senescence. A, Expression of *ANAC017*, *ANAC016*, and core ANAC017-regulated genes during the dark-induced senescence assay. Lowest and highest expression values for each gene have been normalized between 0 and 1. B and C, Expression of *ANAC016*, *ANAC017*, representative ANAC017-controlled genes, and core ATGs based on publically available microarray data (32). NS, natural senescence; expression in leaves with different stages of yellowing (expressed as percentage) during natural senescence; DIS, dark-induced senescence on attached leaves; DET, dark-incubated detached leaves. Heat map color scale represents fold changes normalized to the first time point within each sample group. D, RT-qPCR analysis of *ANAC017* and ANAC017-controlled genes in extended dark-induced senescence time-course. Statistical significance has been shown by two-way ANOVA analysis. Error bars indicate standard upper and lower end point of confidence default interval at 95% (*P* < 0.05; *n* = 3).

Previous research of ANAC017-regulated genes focused on identification of genes that were positively regulated by ANAC017, and could no longer be induced by AA or in double mutant backgrounds of *anac017* with mitochondrial function mutants (Ng et al., 2013; Van Aken et al., 2016a, 2016b). It is also known that upon chemical induction, ANAC017 controlled genes are only highly expressed for a period of time and then return to their initial level (Ng et al., 2013; Van Aken et al., 2016a, 2016b). Therefore, we wanted to see whether we could identify the core ANAC017-regulated genes in one of the described types of dark-induced responses in *ANAC017* overexpressing lines (Figure 3). The vast majority of ANAC017 core genes previously identified, as well as *ANAC016*, were strongly and constitutively induced in both *ANAC017 OE* lines at Day 0. Interestingly, despite these constitutively high expression levels, most of the ANAC017 core target genes (53% in OE3.8 and 63% in OE9) were found in the *transiently upregulated* category (Figure 7A;Supplemental Table S1). This indicates that despite the already high expression levels of ANAC017-dependent genes at Day 0, a very transient senescence-triggered “super-induction” is observed at Day 1, which decreases again by Day 3. In Col-0, no clear induction of ANAC017 target genes was observed on Day 1 of the time course, although *AOX1a* showed a transient dip in expression at Day 1, which was reverted by Day 3.

### *ANAC017* and ANAC017-regulated genes are induced during the later stages of natural senescence

The accelerated senescence phenotype of the *ANAC017 OE* lines raises the question of whether the ANAC017 pathway is also active during senescence in WT plants. Our RNA-seq data set focused on the first 3 d after dark-induction of senescence, and at this stage, the WT plants are still not showing clear visual signs of senescence. As can be seen from Figure 7A, most of the ANAC017-regulon genes are not induced in Col-0a even at Day 3 (Figure 7A;Supplemental Figure S7), with the exception of *UGT74E2* and *AOX1d* that are starting to be induced by Day 3. To assess whether the ANAC017-dependent genes are differentially expressed in WT plants over longer senescence periods, we analyzed their expression in a publicly available microarray time course experiment covering natural senescence, prolonged dark-induced senescence on attached leaves, and dark-incubated detached leaves (Figure 7B; van der Graaff et al., 2006). In line with a positive role of ANAC017 during senescence, many of its classic target genes such as *AOX1a*, *UGT74E2*, *OM66*, and *ANAC013* show a gradual increase in expression during natural and dark-induced senescence. During dark-induced senescence in attached leaves (the condition most similar to our experiments), ANAC017-target genes like *AOX1a* and *ANAC013* are showing sustained induction from Days 6 to 9, and some even earlier at Days 2–4 (e.g. *UGT74E2* and *AOX1d*). In dark-incubated detached leaves, the induction of many ANAC017-dependent genes can be observed as early as two days. Also during natural senescence, ANAC017-dependent genes are gradually but strongly induced, especially when leaf yellowing is becoming visible (Figure 7B).

Interestingly, *ANAC017* and *ANAC016* transcripts themselves are “super-induced” at Day 1 during dark-induced senescence in the overexpression lines (Figure 7A). Moreover, *ANAC017* and *ANAC016* are gradually induced during the later stages of natural senescence in WT plants, peaking when chlorosis can be visually detected (Figure 7C). It can thus be inferred that the ANAC017 pathway is naturally induced during the later stages of senescence.

### Late senescence-induced expression of ANAC017-target genes is repressed in *anac017* knockout lines

Due to a lack of visible differences in leaves upon the induction of senescence between Col-0 plants and both *anac017* lines (Figures 1, B–D and 4), we assessed the expression of ANAC017 target genes during an extended dark-induced senescence time-course up to Day 7 post induction (Figure 7D). Our expression analysis showed that ANAC017-target genes *AOX1a* and *UPOX* showed induced expression during the late stages of senescence (Day 7). In contrast, this induction was repressed in *anac017* knockout mutants, demonstrating that ANAC017 is co-regulating their senescence-induced expression. In agreement, *ANAC017* expression itself is also induced at the late stage of senescence (Day 7). It should be noted that again *ANAC016* expression levels were below the reliable RT-qPCR detection range (Ct > 32) so no consistent differences between Col-0 and *anac017* lines could be observed, confirming *ANAC016* is likely a very minor contributor to overall regulation (Figure 7D).

## Discussion

### Resolving conflicting reports on ANAC016 and ANAC017 function during senescence

Three previous studies have presented ANAC017 as having contrasting roles as positive (Meng et al., 2019) or negative (Kim et al., 2018) regulator of senescence, while Kim et al. (2013) concluded that ANAC017 plays no role in senescence but its closest paralog, ANAC016, is a positive regulator of senescence. It is difficult to explain inconsistent conclusions with regards to the role of ANAC017 during senescence; but differences in growth stages of plants, conditions of their growth and experimental treatments (e.g. detached leaves vs intact plants, dark-induced senescence versus natural senescence) used in these studies are possible factors that could contribute to differences in the observed mutant phenotypes (Supplemental Table S6).

The relationship between the functions and binding sites of ANAC016 and ANAC017 during senescence and mitochondrial signaling has been unclear based on the available literature. As ANAC016 and ANAC017 are the most similar proteins in the Arabidopsis NAC transcription factor family based on sequence similarity, it is likely that they operate in a similar way with regards to their protein activity and which promoters they bind on the genome. This is supported by the fact that ANAC013 and ANAC053 are more divergent in sequence to ANAC017 than ANAC016, and act redundantly to ANAC017 (De Clercq et al., 2013; Van Aken et al., 2016a, 2016b). Notably, there is also a discrepancy in the literature concerning the DNA binding site preference of ANAC016. A previous study suggested the existence of a non-ANAC017 like binding motif (ANAC16BM; GATTGGATTCA; Sakuraba et al., 2015). In contrast, ANAC016 was identified by a yeast one-hybrid screen using the same MDM (CTTGxxxxxCA(A/C)G) from the *AOX1a* and *UGT74E2* promoters that identified ANAC017 and ANAC013 (De Clercq et al., 2013). Furthermore, a genome-wide DNA affinity purification sequencing screen using ANAC016 as bait clearly identified many of the same promoters as ANAC017 and ANAC013, which are part of the ANAC017-controlled MDM regulon (O'Malley et al., 2016). The consensus motif for ANAC016 identified in this DAP-seq experiment is also nearly identical to the ANAC017-binding motif identified by DAP-seq, which was independently confirmed by EMSAs, yeast-one hybrids and Chromatin ImmunoPrecipitation-qPCR (De Clercq et al., 2013; Ng et al., 2013). However the ANAC16BM (Sakuraba et al., 2015) was not found to be enriched in the genome-wide DAP-seq study (O'Malley et al., 2016). Overall, independent studies from two different labs have now shown that ANAC016 binds to the same MDM motif as ANAC017 and ANAC013 using a variety of experimental approaches, whereas ANAC16BM-binding has not been independently confirmed.

With both ANAC016 and ANAC017 implicated in senescence, their individual roles now need to be clarified. Summarizing our findings and those of others, it seems clear that overexpression of ANAC016 or ANAC017 both result in similar fast-senescence phenotypes. In contrast, senescence phenotypes in the *anac016* and *anac017* knockout plants are—at least in our hands—very subtle but display molecular phenotypes associated with dampened senescence-induced expression of ANAC017 target genes in *anac017* mutants (Figure 7D). Although ANAC016 and ANAC017 are very similar in amino acid sequence, there are significant differences in the regulation of their own transcripts. It was previously shown that *ANAC017* transcripts are orders of magnitude more abundant in the cell than *ANAC016* transcripts in young in vitro grown seedlings. (Van Aken et al., 2016a, 2016b) This was also observed in the present RNA-seq and RT-qPCR data sets, where *ANAC017* transcripts are 46 times more abundant than *ANAC016* transcripts at Day 0 in Col-0 (541 versus 12 normalized read counts). Though *ANAC017* transcript abundance is stable during mitochondrial dysfunction (Van Aken et al., 2016a, 2016b), during natural senescence *ANAC017* transcripts are clearly induced toward the later stages, when chlorosis is beginning to be observed. Also *ANAC016* was identified as a senescence upregulated gene with greater than three-fold upregulation from Day 31 after sowing onward (Breeze et al., 2011; Podzimska-Sroka et al., 2015). In our study, *ANAC017* transcripts were stable during the 3 days in Col-0, while *ANAC016* is upregulated ∼4.5-fold at Day 3 in Col-0. Interestingly, *ANAC016* transcripts were already upregulated 5- to 9-fold in the *ANAC017 OE* lines at Day 0. Furthermore, *ANAC016* and *ANAC017* themselves show the same transient “super-induction” up to 50–60x at Day 1 and decline at Day 3, as observed, for example, for *AOX1a* and *UGT74E2*. Here, we showed that *ANAC016* contains two ANAC017 binding motifs in the first intron. Previously published studies showed that such regulation in the first intron can impact on gene expression in a splicing-independent manner (Rose, 2018; Gallegos and Rose, 2019). Similar regulation has also been observed in *C. elegans* (Brabin et al., 2011). Further work would be needed to fully understand the contribution of the identified ANAC017-binding sites in *ANAC016* intron 1 to the regulation of *ANAC016* gene expression by ANAC017 in planta. Due to high similarity between *ANAC016* and *ANAC017*, it is possible that ANAC016 is controlled in a self-amplifying loop as found for ANAC013, for which the promoter can be bound by ANAC017 and ANAC013 (De Clercq et al., 2013). During direct mitochondrial inhibition with AA, the retrograde induction of *AOX1a* is nearly completely abolished in *anac017* mutants, whereas *anac016* mutants respond like WT. This could be easily explained by the much lower abundance of *ANAC016* compared to *ANAC017*, suggesting that ANAC017 is the dominant protein active in the signaling pathway, with smaller contributions of ANAC013 and ANAC016.

**Figure 8:**
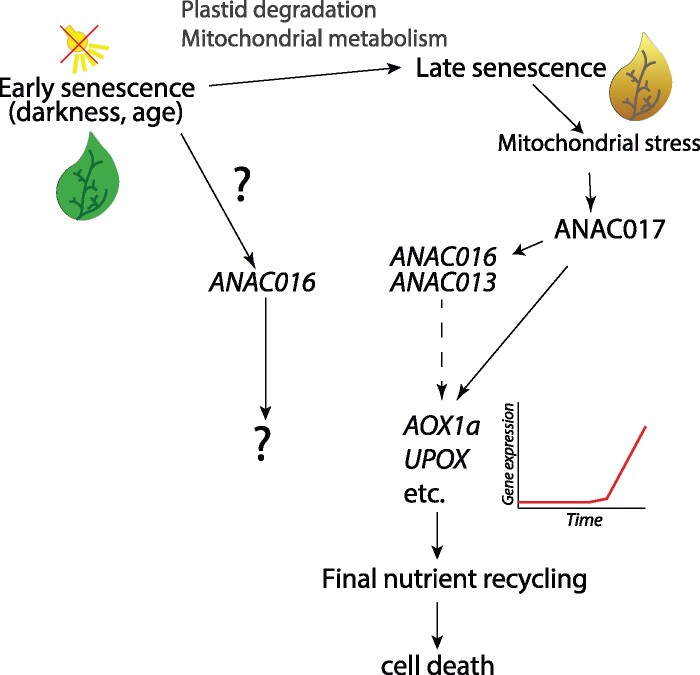
Model of ANAC017 involvement in late senescence. Senescence can be induced by external triggers such as prolonged darkness or by developmental cues such as age. *ANAC016* is induced during early senescence by unknown upstream regulators and has an unclear role. As senescence progresses, plastid, and other cellular components are degraded for remobilization of nutrients, with mitochondria taking an increasingly important part in catabolism. Toward the end of senescence, increased load on mitochondrial function results in activation of the ANAC017 pathway, which transcriptionally upregulates downstream target genes including mitochondrial proteins *AOX1a* and *UPOX*, as well as homologous transcription factors *ANAC013* and *ANAC016* in a feed-forward loop. This may allow mitochondria to continue operating until nutrient recycling is completed and cell death ensues. In *ANAC017* overexpression lines, induced- or natural senescence triggers an exaggerated ANAC017 signaling response, which may trigger the cell into a late-stage senescence-like state and accelerate the senescence program leading to rapid cell death.

Therefore, we propose that ANAC017 is activated transcriptionally and post-translationally during the later stages of leaf senescence, thereby increasing the expression of *ANAC013*, *ANAC016* and the other MDM regulon genes, such as *AOX1a* and *UPOX* (Figure 8). Most likely the activation of ANAC017 is triggered by increasing stress on mitochondrial function during the late senescence process, when mitochondrial metabolic activity is increased and undergoes remodeling to catabolize amino acids and fatty acids, becoming increasingly central hubs of metabolism and energy production (Keech et al., 2007; Chrobok et al., 2016). This may also explain why *anac017* and *anac016* knockout lines have no or perhaps subtle visual phenotypes during senescence, as they only operate toward the end of senescence when leaf yellowing is already visible (Figure 7B). Most likely, the transcript profiles of *ANAC016 OE* and *ANAC017 OE* lines are very similar as they are overexpressing nearly the same protein, and we strongly suspect that the MDM genes are also highly expressed in *ANAC016 OE* lines. Unfortunately, we were not able to obtain the previously published *ANAC016 OE* lines (Kim et al., 2013) to verify this hypothesis.

### *ANAC017* overexpression triggers time-dependent responses in mitochondrial and chloroplast functions

Our RNA-seq analyses identified four different types of responses. Among sustained responses were genes involved in chloroplast biogenesis and function. It was previously shown that chloroplasts are first in line for degradation and recycling processes in order to maintain mitochondrial and cellular function (Peterson and Huffaker, 1975; Avila-Ospina et al., 2014; Chrobok et al., 2016; Law et al., 2018). In the *anac017-1* mutant and Col-0a plants chloroplast-related genes show early type responses, while in both overexpressing lines show a steady (sustained) decline in expression. That is consistent with the observed faster yellowing of leaves in the *ANAC017 OE* lines.

An overrepresentation of genes encoding mitochondrial proteins shows a very different pattern. Several mitochondrial function categories are transiently upregulated in *ANAC017 OE* lines, including mitochondrial organization, transport, and protein import. This indicates that the accelerated senescence is underpinned by a rapid boost in mitochondrial biogenesis/maintenance and activity, most likely to sustain the rapid recycling of nutrients observed in the following two days of the time course. Conversely, mitochondrial functions are also overrepresented in the late down category, indicating that mitochondrial functions are among the last to be switched off during the final moments before complete senescence and cell death. This is in line with previous observations that transcripts encoding mitochondrial proteins involved in primary metabolism and amino acid metabolism are upregulated during the last stages of natural senescence (Chrobok et al., 2016).

As ANAC017 is a key regulator of mitochondrial function during stresses, and mitochondria are crucial to bring senescence and nutrient recycling to a good end, it is not surprising that the ANAC017 pathway is activated during the later stages of senescence. Presumably, this is needed to deal with the increasing stress that later-stage senescence may pose on plant mitochondria. Additionally, it was proposed previously that the ANAC017 pathway may be involved in the suppression of cell death (Van Aken and Pogson, 2017). It could thus be argued that the ANAC017 pathway initially delays cell death to allow maximum recovery of nutrients. One explanation for the perhaps contradictory accelerated senescence in *ANAC017 OE* lines could be that the high expression of the ANAC017 target genes, which also appears to occur during the last moments of natural senescence, “tricks” the cells into thinking the senescence program is nearly completed and thus leads to early programmed cell death. It is unlikely that *ANAC017* overexpression in itself is a senescence trigger, as SAG genes do not appear to be strongly upregulated at Day 0 in our RNAseq analysis. It seems that the external senescence trigger of darkness is needed (Gregersen et al., 2013), which then results in a much faster progression in the *ANAC017 OE* lines. Such careful balances between survival and cell death can also be seen for instance during other process such as autophagy (Das et al., 2012). How exactly overexpression of *ANAC017* affects the cellular balance, ultimately leading to accelerated senescence will require further work.

A previous report observed transcriptional upregulation of the autophagy pathway in 5-week-old *ANAC017 OE* lines and described it as a constitutive upregulation (Meng et al., 2019). The controlled conditions and extensive time course in our data set allowed us to show that ATG genes are not-constitutively induced in *ANAC017 OE* lines. In contrast, they are increasing in expression in the *ANAC017 OE* lines only after a senescence-inducing trigger, leading to *sustained up* type responses in *ANAC017 OE* lines, and most likely contributing to the accelerated senescence and cell death. It will be very interesting to unravel how exactly the ANAC017 regulon can predispose the plants to undergo such fast senescence. The timing of induction of ANAC017-regulated genes during the later stages of senescence matches very closely to the transcriptional upregulation of many ATGs (Figures 6 and 7B; van der Graaff et al., 2006; Broda et al., 2018). This further suggests that in a natural context the ANAC017 and autophagy signaling pathways are synchronized during senescence. Whether there are common signals underlying their activation, or whether one pathway induces the other, is currently unclear.

## Conclusions

In conclusion, our analyses suggest that activation of the ANAC017-pathway is a naturally occurring phenomenon during the later stage of senescence (Figure 8). From a regulatory perspective, it appears that ANAC017 is in fact an upstream regulator of *ANAC016* during late senescence. ANAC016 may then in turn coactivate the same genes as ANAC017, strengthening the induction as observed for ANAC013. As the DNA binding motifs and target genes of ANAC016 and ANAC017 appear to be very similar (De Clercq et al., 2013; O'Malley et al., 2016), the similarity of the fast-senescence phenotypes observed when either gene is overexpressed can easily be explained. Our results also show that overexpression of *ANAC017* and its target genes in itself is not sufficient to accelerate senescence, but that an additional senescence-inducing signal (dark-induction or developmental ageing) is needed to expedite senescence, autophagy, and ultimately cell death. It thus appears that ANAC017 activation predisposes or “primes” plants for accelerated senescence.

## Methods

### Plant material and growth conditions

Arabidopsis, ecotype Col-0 from our lab was used as a control for all experiments and here named Col-0a to distinguish from Col-0 obtained from Kim et al. (2018) and here named Col-0b. *anac017EMS* was previously published by Ng et al. (2013); *OEa* was previously published by Van Aken et al. (2016a, 2016b); a*nac017-1* and *ANAC017 OX* line were obtained from Kim et al. (2018); a*nac016* (SALK_074316) was obtained from Arabidopsis Biological Resource Center. *OEb* and *OEc* were generated in Col-0a by floral dipping using 35S expression vector pB7WG2 (Karimi et al., 2002). All plants were stratified for 3 days in 4°C and grown for 5 weeks in soil consisting of soil, perlite, and vermiculate mixture in 4:1:1 ratio under LD conditions (16 h light, 8 h darkness, 100 µmol m^−2^ s^−1^) at 22°C. The plants had started flowering by the start of the dark-induced senescence assays.

### AA treatment

AA treatment was performed as previously described (Broda and Van Aken, 2018). In general, 50 µM AA was used to spray 2-week-old seedlings from a distance of about 20 cm using spray bottle. Samples were collected at indicated time-points, snap frozen in liquid nitrogen, and stored at −80°C until use.

### RNA extraction, cDNA synthesis, RT-qPCR

Tissue subjected for analysis were snap-frozen in liquid nitrogen (*n* = 3) and stored at −80°C until use. Tissue was then ground using a bead mill and total RNA was isolated using Spectrum^TM^ Plant Total RNA kit (Sigma-Aldrich, STRN250-1KT) with On-Column DNase treatment (Sigma-Aldrich, DNASE70) following the manufacturer’s instructions; 500 ng of total RNA was used for cDNA synthesis using iScript cDNA synthesis kit (Bio-rad, 1708890; Broda and Van Aken, 2018). cDNA was further diluted and used for quantitative real-time PCR using QuantiNova SYBR green PCR kit (Qiagen, 208056). A list of primers used for the RT-qPCR can be found in Supplemental Table S5. Two housekeeping genes were used for normalization: *UBQ10* and *AKT2* (Czechowski et al., 2005) and analyzed using geometric averaging of multiple control genes (Vandesompele et al., 2002).

### Senescence assay

Plants were grown for 5 weeks in soil in standard LD growth conditions. Plants were randomized throughout the tray, shelf position, and room position to compensate for environmental factors. Leaf 5 was collected for starch and *ANAC017* and *ANAC016* gene expression analysis. Leaves 6 and 7 were used for chlorophyll and ion leakage analysis.

For starch analysis, leaf 5 was collected at the end of day and end of night time point and tissue was snap frozen in liquid nitrogen and kept at −80°C until further use. Tissue was ground using a bead mill and incubated with 80% ethanol at 90°C for 3 min with maximum agitation. Samples were then centrifuged for 10 min at 20,000*g*. This step was repeated three times. The pellet was then used for starch enzymatic assays, using a protocol described previously (Smith and Zeeman, 2006; O'Leary et al., 2017).

Leaves 6 and 7 were covered with aluminum foil for dark-induced senescence and harvested at indicated time points. On each day, pictures were taken of all leaves using the same camera objective and automatic exposure. For illustration purposes, representative leaves were selected using the Quick Selection tool in Photoshop and placed on a uniform black background. After pictures were taken, chlorophyll was measured using a SPAD meter (SPAD-502Plus, Konica Minolta). Each leaf was measured multiple times in different positions on the leaf blade. Leaves 6 and 7 were then combined for ion leakage measurement. Ion leakage was described previously (Guo and Gan, 2006) and applied with minor changes. Leaves were cleaned of soil residues, placed in 50-mL falcon tube with 20 mL dH_2_O, and placed on a rocker for 30 min with gentle rocking. Initial measurement was made using a HI98192 meter (Hanna Instruments). Leaves were then boiled and cooled down to room temperature and a second measurement was taken. Ion leakage is presented as a percentage of initial over the final measurement.

### RNA-seq library preparation and differential gene expression analysis

Individually darkened leaves 6 and 7 from Days 0, 1, and 3 were collected and pooled together from Col-0 a, anac017-1 (SALK), OE3.8, and OE9 from senescence assay for total RNA isolation using same procedure as described above.

For RNA-seq library preparation, total RNA was treated with Ambion Turbo DNase (ThermoFisher Scientific, AM1907) and quantified using Qubit RNA BR Assay Kit (Invitrogen, Q10210); 500 ng of RNA was then used for library preparation using TruSeq Stranded Total RNA with Ribo-Zero Plant Kit (Illumina, RS-122-2401) and TruSeq RNA UD Indexes for up to 96 samples (Illumina, 20022371). Samples were sequenced using a HiSeq1500 with SBS kit v3 for 50 cycles (Illumina, FC-401-3002). Alignment of reads was performed against the TAIR10 annotation using STAR (Dobin et al., 2013). On average 24 million reads per sample were mapped (Supplemental Table S2). Counts were assigned to genes using summarized overlaps and analysis of DEGs was performed with the EdgeR package (Robinson et al., 2010; McCarthy et al., 2012). Transcripts were considered differentially expressed if FDR ≤ 0.05 and fold change ≥ 2 or fold change ≤ −2. Raw RNA-seq data files are available from ArrayExpress with accession number E-MTAB-8478.

Enrichment of GO analysis was performed using AgriGO v2 (Tian et al., 2017). Graphical representation of GO terms was performed using the GOplot package in R (Walter et al., 2015).

### Electromobility shift assays

*ANAC017* lacking its C-terminal transmembrane domain was cloned into pDEST15 GST-fusion vector and recombinantly expressed and purified in Rosetta 2 (DE3) cells as previously described (Ng et al., 2013). Oligonucleotide probes (Supplemental Table S5) were annealed and phosphorylated using NEB T4 polynucleotide kinase with gamma ^32^P-ATP (Perkin Elmer) and purified using G-25 Sepharose Quick spin columns (Roche). Binding reactions were performed as previously described (Ng et al., 2013), separated on 12% (w/v) acrylamide 0.5 × TBE gels, dried and imaged using a phosphorimager.

## Accession numbers

RNA-seq data files are available from ArrayExpress with accession number E-MTAB-8478. Gene accession numbers used in this study: ANAC017 (At1g34190), ANAC016 (At1g34180), AOX1a (At3g22370), SAG12 (At5g45890), YSL7 (At1g65730), YLS9 (At2g35980), UPOX (At2g21640), and UGT74E2 (At1g05680).

## Supplemental data

The following materials are available in the online version of this article.

**Supplemental Figure S1**. Functional comparison of ANAC017 and ANAC016 in mitochondrial retrograde signaling responses.

**Supplemental Figure S2**. ANAC017 and ANAC016 binding motifs.

**Supplemental Figure S3**. Two-way ANOVA test significance in senescence assay.

**Supplemental Figure S4**. Accelerated senescence of detached dark-incubated leaves.

**Supplemental Figure S5**. Extended dark-induced senescence time-course on Col-0a, *anac017EMS*, *anac017-1*, and *anac016*.

**Supplemental Figure S6**. Expression levels of hormone related genes during senescence.

**Supplemental Figure S7**. Expression of ANAC017 controlled genes over the dark-induced senescence time-course in Col-0a plants.

**Supplemental Table S1**. RNA-seq data for Col-0a, *anac017-1*, *ANAC017 OEa*, and *OEc* during dark-induced senescence time-course.

**Supplemental Table S2**. Sums of counts of technical replicates obtained for each of the biological samples analyzed by RNA-seq.

**Supplemental Table S3**. GO Term analyses of DEGs in different types of time-dependent responses in dark-induced senescence time-course for Col-a, *anac017-1* and commonly expressed genes between *ANAC017 OEa* and *OEc*.

**Supplemental Table S4**. GO Term analyses of DEGs in different types of time-dependent responses in dark-induced senescence time-course for commonly expressed genes between *ANAC017 OEa* and *OEc*.

**Supplemental Table S5**. Primer sequences used in the RT-qPCR and EMSA analyses.

**Supplemental Table S6**. A summary of experimental design used in previous studies concerning role of ANAC017 in senescence.

## Supplementary Material

kiab195_Supplementary_Data

## Notes

Increased expression of a transcription factor that mediates mitochondrial signaling primes plants for accelerated senescence, induction of autophagy, and cell death.

M.B., A.P., A.H.M., and O.V.A. designed experiments. M.B., A.P., O.V.A., K.K., and B.O. performed experiments. M.B., O.V.A. and B.O. analyzed data. M.B., C.P.L., O.V.A., and A.H.M. wrote the manuscript with input from the coauthors.

The author responsible for distribution of materials integral to the findings presented in this article in accordance with the policy described in the Instructions for Authors (https://academic.oup.com/plphys/pages/general-instructions) is: Olivier Van Aken (olivier.van_aken@biol.lu.se).
