# Supplementary material for: Increased expression of ANAC017 primes for accelerated senescence
Source: Plant Physiol. 2021 Apr 29;186(4):2205–21. doi: 10.1093/plphys/kiab195 (PMC8331134; doi:10.1093/plphys/kiab195)
Supplement: kiab195_Supplementary_Data [file kiab195_supplementary_data.zip › pp.00230.2021-s01.pdf]

A

```
NAC016_At1g34180      MVDSSRDSCFKAGKFSAPGFRFHPTDEELVVYYLKRKICCKKLRVNAIGVVDVYKVDPSE      60
NAC017_At1g34190      MADSSPDSCFKGGKFSAPGFRFHPTDEELVMYYLKRKICRKRLRVNVIGVVDVYKMDPEE      60
                      *.*** *****.******************:******** *:****.********:**.*

NAC016_At1g34180      LPGNFQHLLIDFDSCLSMLKTGDRQWFFFTPRNRKYPNAARSSRGTATGYWKATGKDRVI      120
NAC017_At1g34190      LPGQ------------SMLKTGDRQWFYFTPRSRKYPNAARSNRGTENGYWKATGKDRVI      108
                      ***:            ***********:****.*********.*** .************

NAC016_At1g34180      EYNSRSVGLKKTLVFYRGRAPNGERTDWVMHEYTMDEEELGRCKNAKEYYALYKLYKKSG      180
NAC017_At1g34190      EYNSRSVGLKKTLVFYRGRAPSGERTDWVMHEYTMDEDELGRCKNPQEYYALYKLFKKSG      168
                      *********************.***************:******* :********:****

NAC016_At1g34180      AGPKNGEQYGAPFQEEEWVDSDSEDADS--VAVPDYPVVRYENGPCVDDTKFCNPVKLQL      238
NAC017_At1g34190      AGPKNGEQYGAPFQEEEWVDDDNEDVNAIAVAVPEQPVVRYEDARRVDERRLFNPVILQL      228
                      ********************.*.**.::  ****: ******:.  **: :: *** ***

NAC016_At1g34180      EDIEKLLNEIPDAPGVNQRQFDEFVGVPQGNSAEVIQSTLLNNSSGEYIDPRTNGMFLPN      298
NAC017_At1g34190      EDIDELLNGIPNAPGVPQRC------IPQVNSEEELQSTLVNNSAR---------EFLPN      273
                      ***::*** **:**** **       :** ** * :****:***:           ****

NAC016_At1g34180      GQLYNRDSSFQSHLNSFEATSGMAPLLDNEKEEYIEMNDL-LIPELGASSTEKSTEFLNH      357
NAC017_At1g34190      GQQYNRPSSFDSLET----AEVTSAPLVFEKEDFIEMDDLLLIPEFGASSTEKAAQFSNH      329
                      ** *** ***:*  .    :.  :  *  ***::***:** ****:*******:::* **

NAC016_At1g34180      GEFGDVNEYDQLFNDISV-------FQGTSTDLSCLSNFTNNTSGQRQQLLYEQFQYQTP      410
NAC017_At1g34190      GEFDDFNEFDQLFHDVSMSLDMEPIDQGTSANLSSLSDSANYTSDQKQQLLYQQFQDQTP      389
                      ***.*.**:****:*:*:        ****::**.**: :* **.*:*****:*** ***

NAC016_At1g34180      ENQLNNYMHPSTTLNQFTDNMWFKDDQAALYVQPPQSSSGAFTSQSTGVMPESMNPTMSV      470
NAC017_At1g34190      ENQLNNIMDPSTTLNQITSDIWFEDDQAILFDQ-QQSFSGAFASPSSGVMPDSTNPTMSV      448
                      ****** *.*******:*.::**:**** *: *  ** ****:* *:****:* ******

NAC016_At1g34180      NPQYKEGQNGGGTRSQFSSALWELLESIPSTPASACEGPLNQTFVRMSSFSRIRFNGTSV      530
NAC017_At1g34190      NAQGHEIQNGGGTTSQFSSALWALMDSIPSTPASACEGPLNRTFVRMSSFSRMRFNGKAN      508
                      * * :* ****** ******** *::**************:**********:****.:

NAC016_At1g34180      T-SRKVTVAKKRISNRGFLLLSIMGALCAIFWVFKATVGVMGRPLLS-- 576
NAC017_At1g34190      GTPVSTTIAKKGIRNRGFLLLSIVGALCAIFWVLVATVRVSGRSLLLKD 557
                          ..*:*** * *********:*********: *** * ** **
```

B

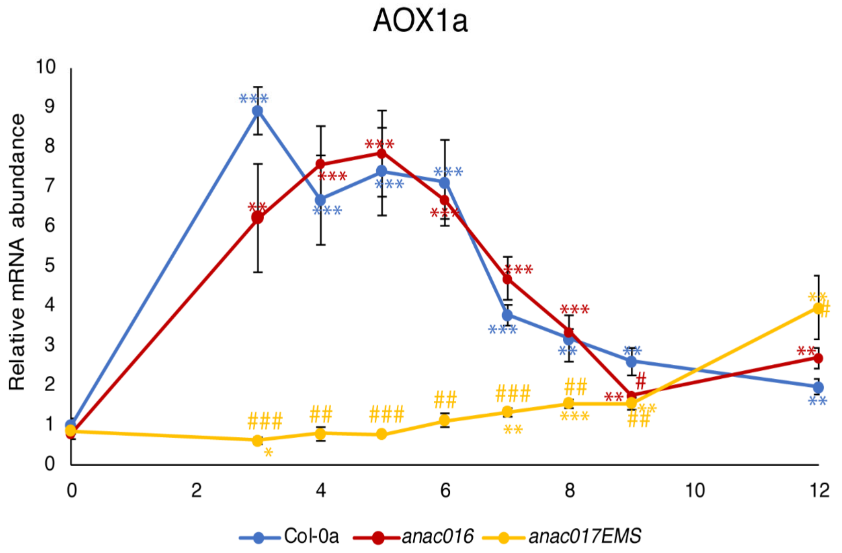

Supplemental figure S1. Functional comparison of ANAC017 and ANAC016 in mitochondrial retrograde signalling responses. A. Protein sequence alignment of ANAC017 and ANAC016. B. Expression of *AOX1a*, a marker for mitochondrial retrograde signalling, during Antimycin A time-course. Student's t-test has been used to verify statistical significance. Error bars represent standard error (n=3). Asterisks (*) represent a statistical significance of a genotype in comparison to its own 0 time point. Hash sign (#) represents statistical significance of genotype at indicated time point compared to same time point in Col-0. * or # p≤0.1, ** or ## p≤0.05, *** or ### p≤0.01.

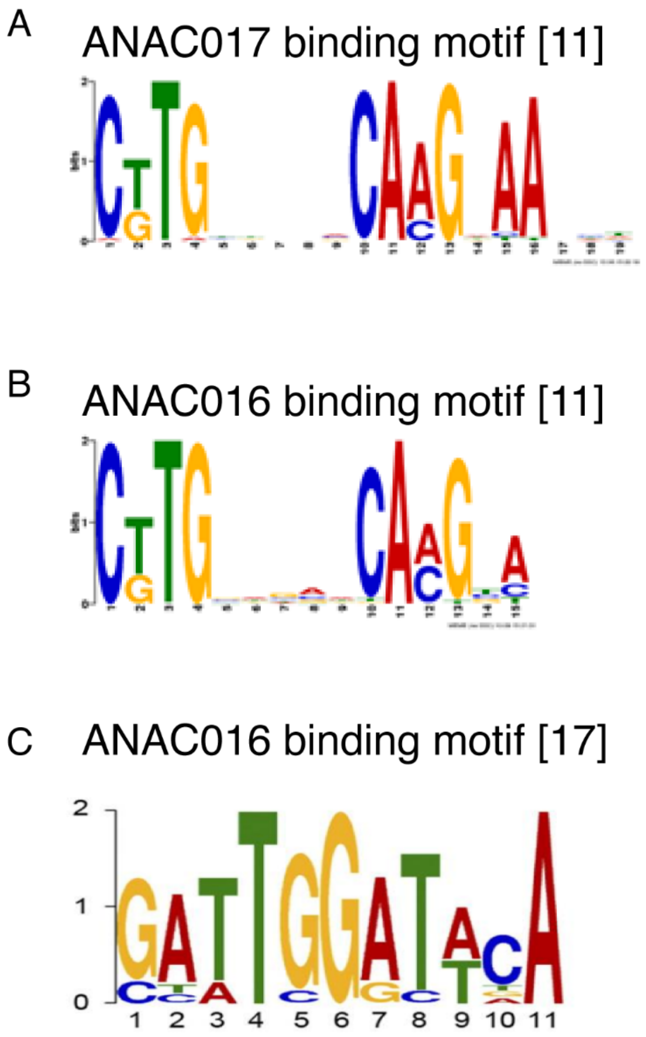

Supplemental figure S2. ANAC017 and ANAC016 binding motifs. A. ANAC017 binding motif based on DAP-Seq analyses (O'Malley et al., 2016). B. ANAC016 binding motif from DAP-seq analyses (11). C. ANAC016 binding motif as found by Sakuraba et al., 2015. Image taken from Sakuraba et al., 2015.

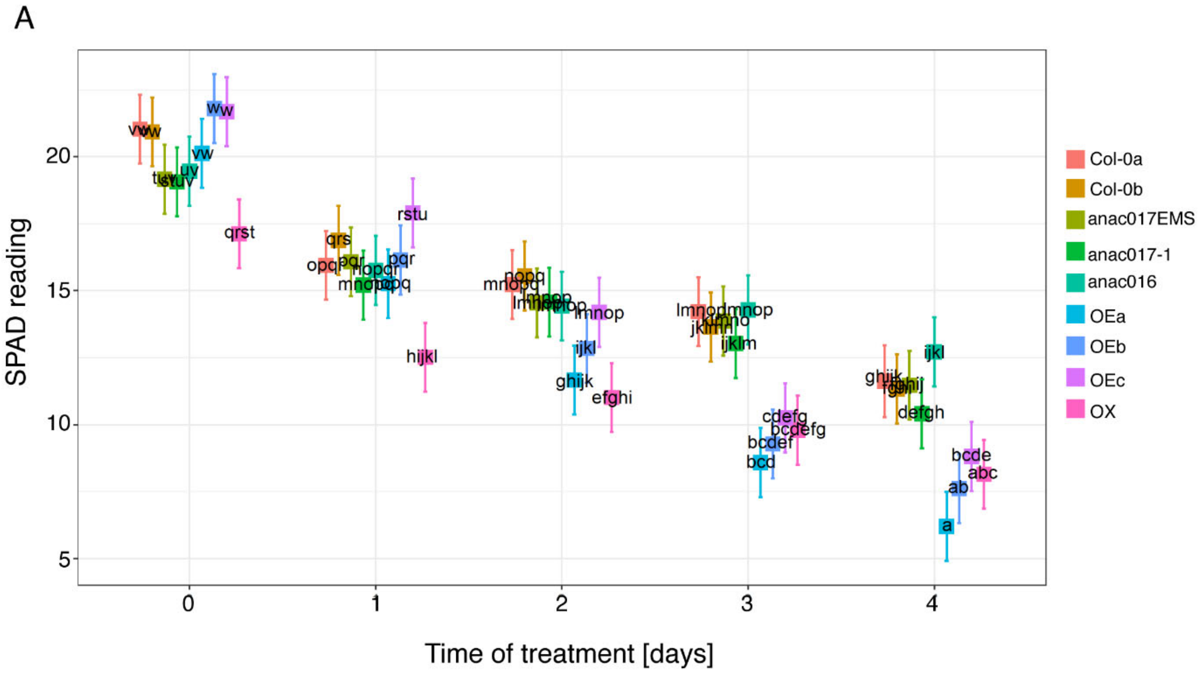

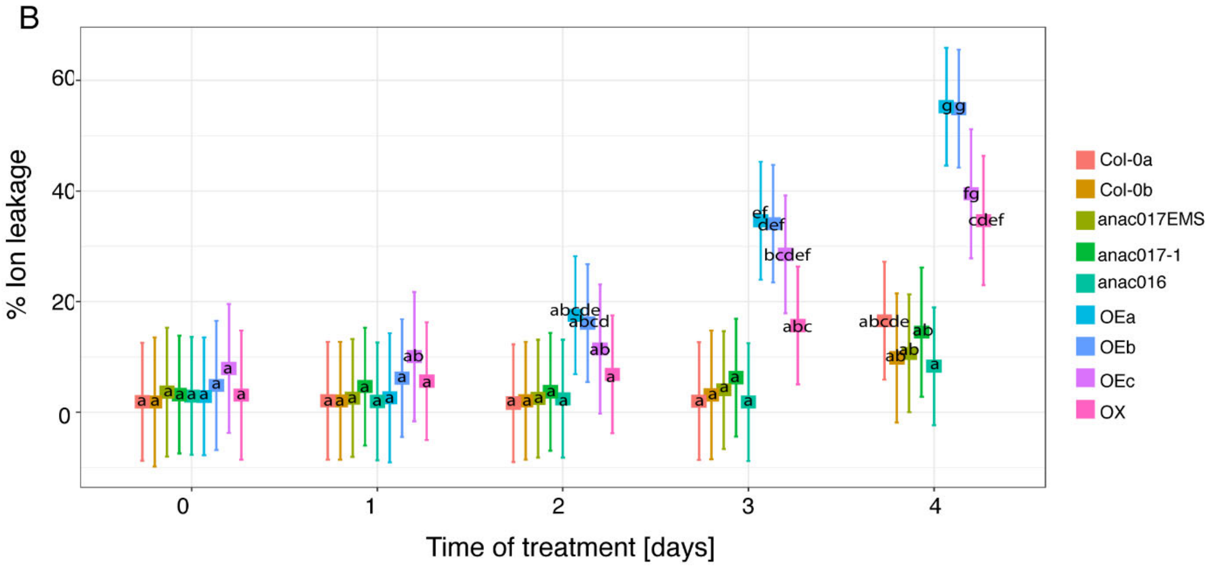

Supplemental figure S3. Two-way ANOVA test significance in senescence assay. A. Two-way ANOVA significance of SPAD chlorophyll measurements taken over the course of 4 days of dark-induced senescence. B. Two-way ANOVA significance of ion leakage measurements taken over the course of 4 days of dark-induced senescence. Error bars represent upper and lower end point of confidence default interval at 95% ($p<0.05$; $n=6$)).

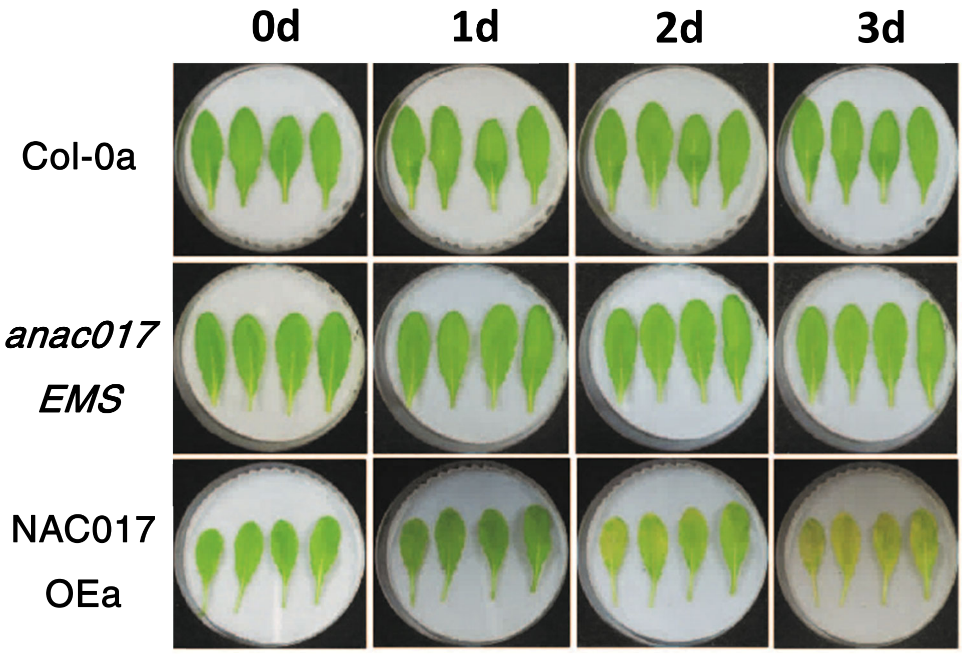

Supplemental figure S4. **Accelerated senescence of detached dark-incubated leaves in *ANAC017 OE* lines** compared to Col-0 and *anac017EMS* mutant line.

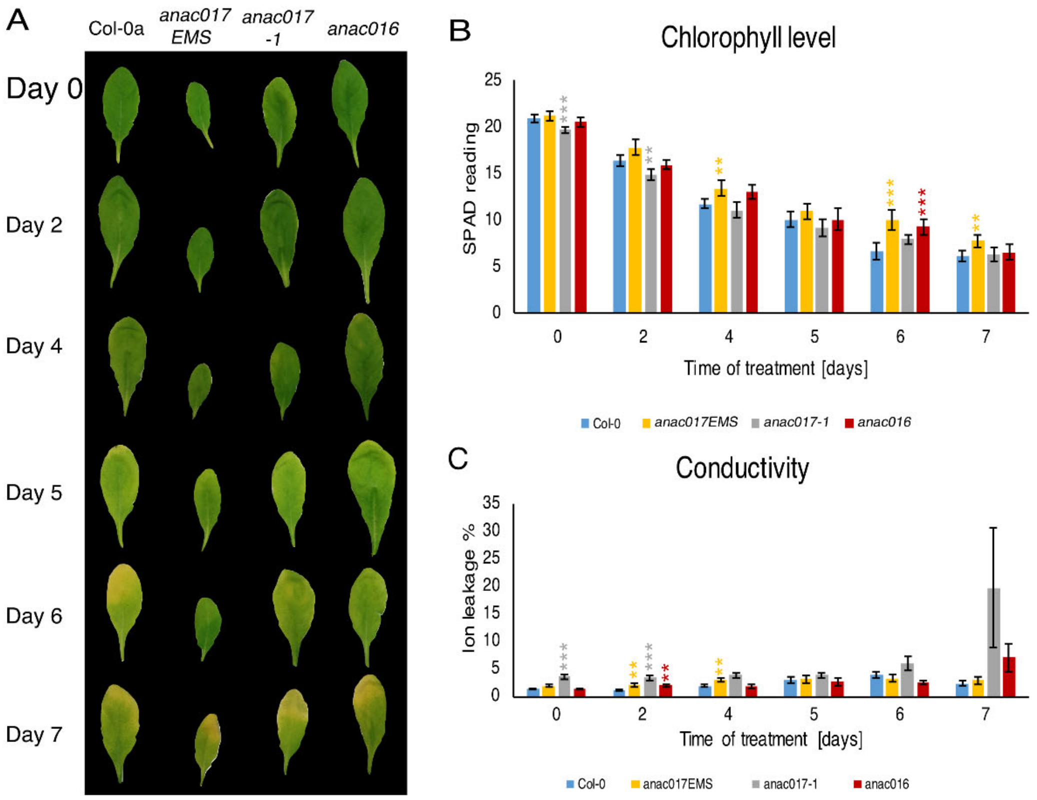

Supplemental figure S5. Extended dark-induced senescence time-course on Col-0a, *anac017EMS, anac017-1 and anac016*. A. Phenotype of analysed genotypes in individually darkened leaf senescence time-course at indicated time points. For representation purposes, leaves were digitally extracted and placed on a black background. Chlorophyll level (B) and ion leakage (C) of leaves during extended senescence time-course at indicated time-points. * $p \leq 0.1$, ** $p \leq 0.05$, *** $p \leq 0.01$, according to student's t-test. Error bars represent standard error (n=3).

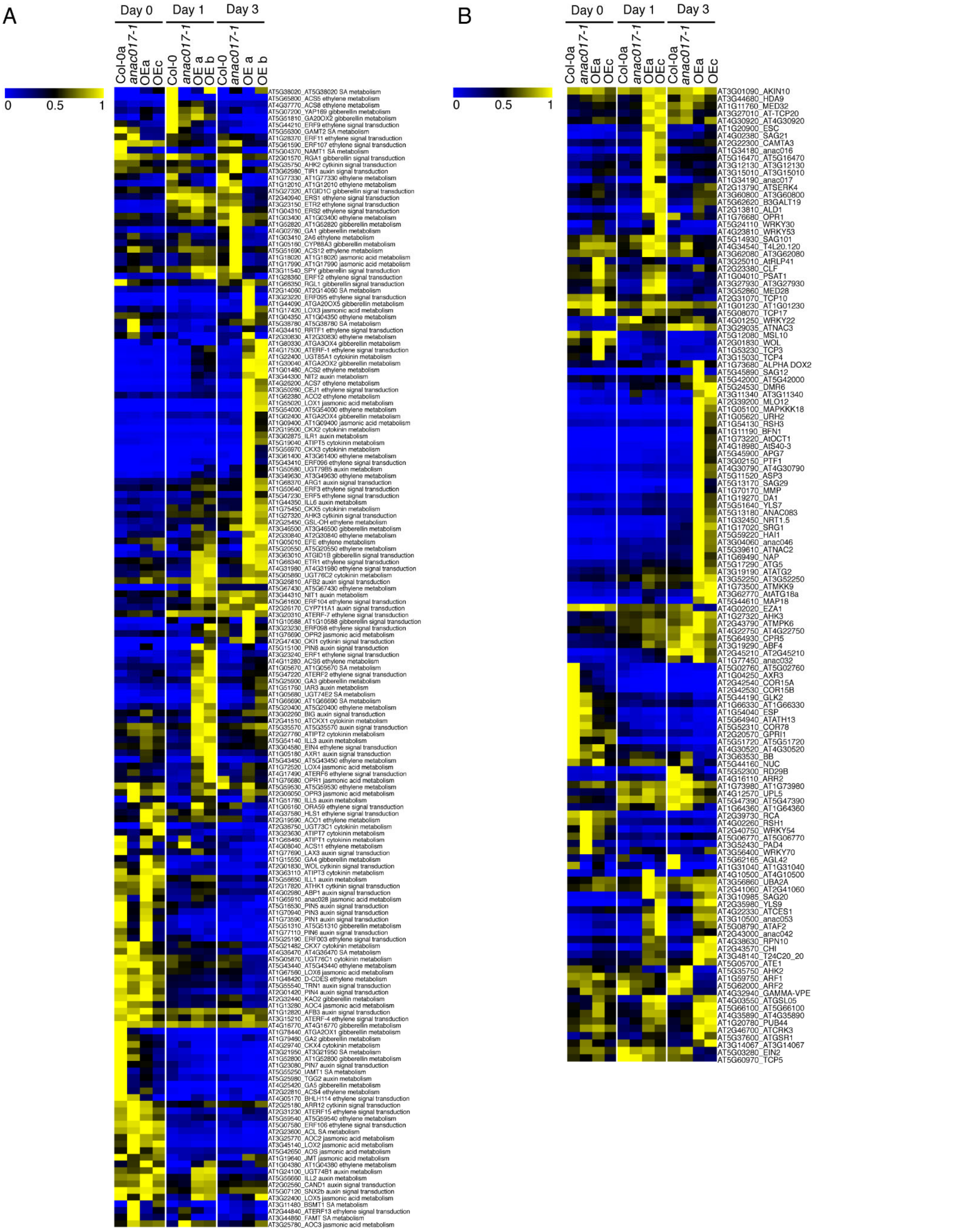

Supplemental figure S6. Expression levels of hormone related genes during senescence. A heat map of A. DEGs involved in biosynthesis, degradation and signalling of ethylene, salicylic acid (SA), jasmonic acid (JA), gibberellins, auxins and cytokinins. B. DEGs previously published

to be involved in senescence. The color bar represents normalised gene expression between 0 and 1, with 1 set as the maximum value for that gene across all samples.

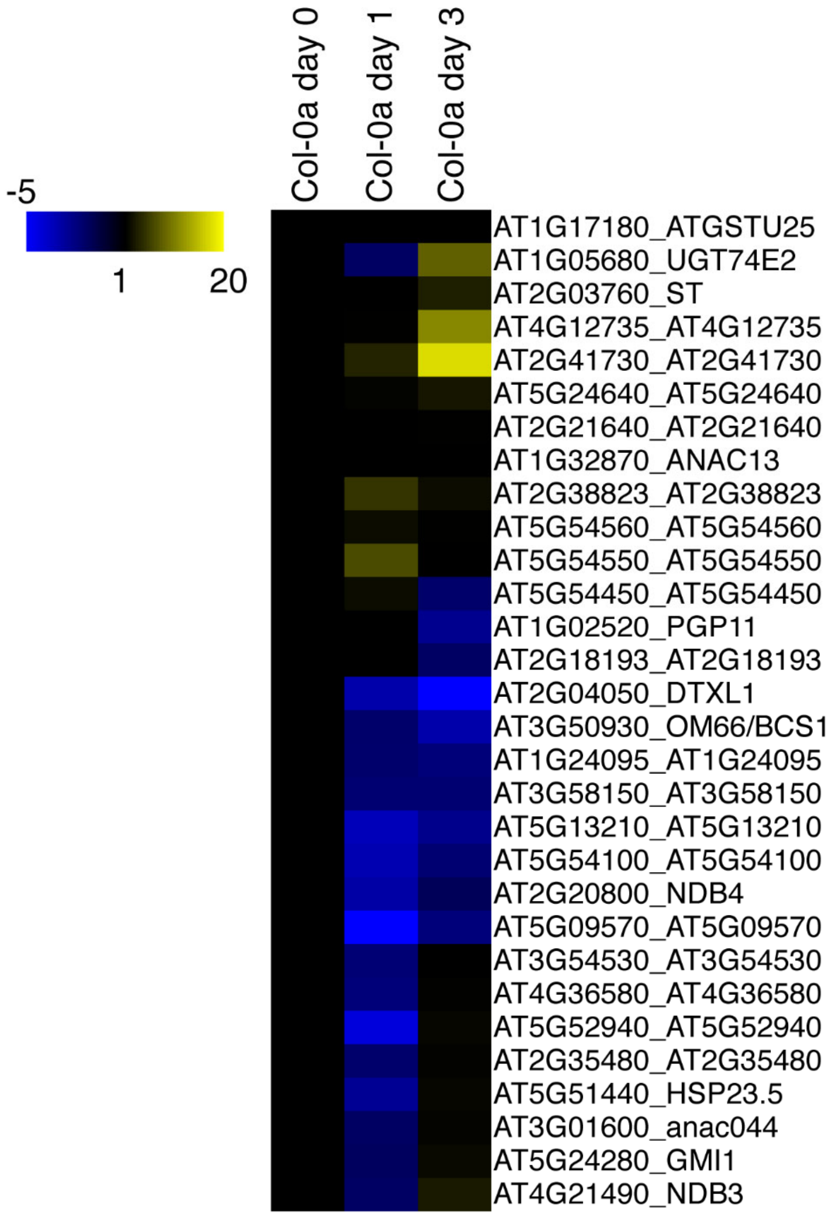

Supplemental figure S7. Expression of ANAC017 controlled genes over the dark-induced senescence time-course in Col-0a plants. The color bar represents normalised fold change gene expression with Col-0 Day 0 set as 1.
